# Case Study of a Rare Genetic Disorder: Congenital Insensitivity to Pain With Anhidrosis

**DOI:** 10.7759/cureus.12984

**Published:** 2021-01-29

**Authors:** Saqib M Mughal, Ayaaz Farhat

**Affiliations:** 1 Family Medicine, Alpha Medical Practice, Birmingham, GBR; 2 Family Medicine, Primary Health Care Corporation, London, GBR

**Keywords:** autosomal recessive disorder, congenital insensitivity to pain, lack of pain sensation

## Abstract

A rare autosomal recessive disorder, congenital insensitivity to pain with anhidrosis, is characterised by the congenital lack of pain sensation. Other characteristic symptoms include no sweating, recurrent episodes of hyperpyrexia, retardation of mental abilities and self-mutilating behaviour. Herein, we present a case of a one-year-old male child who initially presented with self-bites on the tongue and then multiple fractures with no report of pain or crying, which initially indicated carelessness of parents. Based on further in-depth assessment indicating a family history of similar weak bones and no pain, the paediatric team conducted investigations along with genetic tests. The child was diagnosed with congenital insensitivity to pain with anhidrosis. Another sibling born later also had the same disorder. Both the children developed eczema, which was difficult to cure due to constant scratching by children as they did not feel any pain. Follow-up studies indicated a slight difficulty in learning abilities and delay in the achievement of milestones. This case report indicates the need for rigorous investigations in such cases to understand the aetiology and appropriate counselling of parents for the utmost care of the child.

## Introduction

This is a case report of presumed non-accidental injury that had an underlying medical condition of congenital insensitivity to pain with anhidrosis (CIPA). CIPA is a rare autosomal recessive genetic disease caused by certain gene mutations. CIPA, also known as hereditary sensory and autonomic neuropathy type IV, is a rare genetic condition [1,2]. Very few individuals are affected by this genetical disorder, but cases can be found worldwide. CIPA is clinically characterised by the ability to feel a given stimulus but the inability to perceive pain along with anhidrosis. Insensitivity to pain and thermal sensation, no sweating (anhidrosis) and mental retardation/distress are three major clinical findings associated with CIPA. Various other sensations, like touch and pressure, are maintained. This insensitivity towards pain and thermal sensations could lead to multiple bone fractures, burns and sometimes self-mutilation of fingers, tongue and lips.

Genetic loss-of-function mutation in human tropomyosin receptor kinase A (TRKA gene NTRK1) encoding receptor tyrosine kinase for a nerve growth factor (NGF) was reported to be the cause for CIPA [3]. Nociceptive sensory neurons and sympathetic autonomic neurons are under the surveillance of NGF and play a role in the activation and homeostasis of other cell types [4]. Thus, the mutation in NTRK1 causes deficient development of the somatic sensory system, which is located in dorsal root ganglion sensory neurons for pain and temperature [5]. Development of the autonomic sympathetic nervous system is also affected and results in lost innervation of sweat glands by the sympathetic nervous system [6]. Central nervous system and bidirectional communication between immune system and nervous system are also affected by NTRK1 mutation [7]. In addition, functional alterations in NGF affect the normal procedure of fracture consolidation [8]. Differentiation of osteoblast/osteoprogenitor cells is also hindered. Due to the lack of nociceptive fibres in the skeletal system, metabolism of bones is also affected, and bone fractures are recurrent and common in CIPA patients due to the absence of the trophic role of nociceptive fibres [9].

Few laboratory tests along with clinical analysis are conducted for diagnosing CIPA based on symptoms like pain insensitivity, anhidrosis, and mental distress. Some tests, like axonal flare test and biopsies, are also performed. In flare test on injection of histamine under the skin, normal flare is not caused at the site of injection in CIPA patients [10,11]. Reduced number of myelinated and unmyelinated fibres of small diameter and normal count of large-diameter fibres are observed in sural nerve biopsy in CIPA patients [12]. However, the molecular test for the evaluation of mutation in NTKR1 is considered the confirmatory test for diagnosis of CIPA, but its availability is quite challenging.

In this study, we report a case of a one-year-old child suffering from CIPA, including the course of investigations and follow-up.

## Case presentation

A one-year-old male child was brought to our primary care clinic by his mother. She stated that he had been teething for some time now and was biting and causing damage to his tongue. The child was examined, and healed lacerations were observed on the tongue. The child was also examined by the dental team, which commented that tongue biting could be a normal behaviour at this age.

The child presented to the clinic along with parents after approximately 14 months with swelling on his left arm. Parents visited the local emergency department and reported that they were not aware of any injury and had just observed the swollen arm of the child. There was no report of pain or crying observed in the child. On examination, the child was found to have a few-days-old fracture of ulna. Since no clear cause of the injury was found and the parents could not provide any explanation for the same, the protocol for the treatment of non-accidental injury was followed by the hospital. Due to the south Asian origin of parents and their poor English-speaking ability, there were some communication difficulties. They reasoned that since the child did not show any sign of distress, they were not aware of the injury and had not visited the doctor earlier. The hospital team became suspicious about the parents’ behaviour and unawareness and was not convinced by the narration of parents. The child was put under a safeguarding order, which meant a full detailed review was deemed necessary, and his inpatient medical investigations and treatment were initiated. During the course of the investigations, which included skeletal surveys, another healed fracture was found in the right lower leg. It was intimated to the parents that the child would not be discharged back to them until a complete investigation has been conducted. The parents were distressed and visited their family physician as they could not understand the safeguarding procedure. The child’s father informed the family physician (who communicated with them in their native language) that some family members in their home country have a similar problem of weak bones with no pain sensation. This information was communicated to the paediatric team, who accordingly conducted further investigations. These included genetic testing, which led to the diagnosis. The child was diagnosed with CIPA and was examined by various specialists. The child was eventually allowed to go home with the parents and continued to have follow-ups. The parents were found to have a consanguineous marriage, which could be the reason for recessive syndromes. They were recommended to consult with a genetic counsellor. Eighteen months after the diagnosis, the couple had a second child (female), who was also diagnosed with the same disorder. Follow-up of both children was conducted for the following five years and they were found to have mild learning difficulties and had delayed achievement of their milestones. Due to the history of multiple fractures, the children were at risk of developing Charcot joints. The male child had eczema, which was difficult to treat due to continuous scratching by the child since he did not have any sensation for pain. This caused significant damage to his skin. The younger sibling faced similar issues and had multiple fractures and eczema. There was some discussion with the family about testing the rest of the extended family who may have exhibited similar symptoms but this was complicated with the members living abroad.

## Discussion

Congenital insensitivity to pain has been classified as an autosomal recessive genetical disorder that is also termed as hereditary sensory and autonomic neuropathies. It can be categorised into five sub-types based on the stage of onset, additional symptoms and genetic mutations responsible for it [1]. It is caused by the mutation in NTRK1 gene, which results in failed differentiation and migration of neural crest cells. This leads to the total absence of small-diameter myelinated and unmyelinated nerve fibres, thereby causing loss of pain and temperature sensation. Moreover, innervation of sweat glands is also absent, causing anhidrosis [13]. Various symptoms associated with CIPA include congenital analgesia, self-mutilation/injury, multiple fractures, Charcot joints and anhidrosis [14]. There is also some association with mental retardation [15,16]. Reports have also suggested hyperhidrosis and impaired lacrimation associated with CIPA. Failure to thrive associated with insufficient weight gain in the child, along with repeated episodes of fever secondary to anhidrosis, were other symptoms reported in the case of CIPA [17].

In many reported cases of CIPA, the affected child is from consanguineous parents [18]. Similar to these cases, the marriage of the parents in this study was reported as consanguineous. Moreover, there was also some positive family history of similar conditions within the extended family. The child in our case was diagnosed at an early age with CIPA by the genetics team. Initially, the case presentation was confused with non-accidental injury and led to the separation of child from his parents. The parents were subjected to a child protection enquiry whilst the medical investigations were undergoing. This case was further complicated by poor communication and language difficulties of the parents. It is apparent from this case how such conditions could be confused with on-accidental injury. As the child grew up, it became increasingly difficult for the parents since there was a constant need for proper vigilance of the child to prevent injuries and self-mutilation. Mental health of the mother suffered from a continuous toll and pressure as any undesirable incident would cause a guilt in her for negligence in her duty towards the child. Other evidences also suggest that chronic conditions in children who require intense requirement of care have a negative effect on paternal mental health with the feelings of guilt or sorrow. It was also apparent during the course of follow-up that the child did not have any fear for painful stimuli as he would just jump off a chair or stairs, etc.

Treatment options

The treatment of CIPA is symptomatic and requires a multidisciplinary approach, incorporating both the physical and mental health issues of both patients and caregivers. Early diagnosis of disease during infancy could make parents more aware of risk factors, and thus, the accidents could be avoided with a constant awareness of the child’s activities. Special dental treatments have been recommended to avoid the severe outcomes due to self-mutilation by the child. Extraction of primary teeth of children with CIPA was recommended in the 1960s for avoiding self-mutilation. It was suggested to place both upper and lower full dentures. However, tooth extraction is an extreme step, and parents are usually reluctant to proceed with it [19]. Better options are now available that are more suitable to the quality of life of the CIPA child, such as wearing of dental guards along with strict vigilance by the parents. A team of multidisciplinary physicians along with dentists is needed for prevention of self-mutilation in CIPA patients.

There are other manifestations as well that are important and need to be considered. Anhidrosis in CIPA patients could cause recurrent fever and could be fatal if not diagnosed early. In addition, a high prevalence of infections has been observed. Skin conditions such as dermatitis and deep bone infections are other common issues associated with CIPA, and the most involved pathogen is *Staphylococcus aureus*. Antibiotic resistance observed in these patients limits the treatment options [20].

## Conclusions

This interesting case, which was brought to the family medicine clinic, brings about many learning points. There was an importance in thorough history taking, including family history, which was vital in reaching a correct diagnosis. In areas where consanguineous relationships are common, healthcare professionals need to keep these factors in mind as it can mean an increased possibility of genetic conditions. If not diagnosed correctly, this can lead to not only a delay in appropriate management but also the consequences of an incorrect diagnosis, for example safeguarding concerns that came to light in this particular case. 

Genetic conditions, such as CIPA, manifest in childhood and therefore not only affect the child but also have a massive effect on the parents. They often require support for themselves due to the high level of complexities in managing the patient. Due to the nature of the condition, the parents need to be well educated in the management of the condition. Overall these types of conditions can be challenging for clinicians along with parents and require a multidisciplinary approach to enable the best outcomes.
